# Reparametrization-based estimation of genetic parameters in multi-trait animal model using Integrated Nested Laplace Approximation

**DOI:** 10.1007/s00122-015-2622-x

**Published:** 2015-11-18

**Authors:** Boby Mathew, Anna Marie Holand, Petri Koistinen, Jens Léon, Mikko J. Sillanpää

**Affiliations:** Institute of Crop Science and Resource Conservation, University of Bonn, 53115 Bonn, Germany; Department of Mathematical Sciences, Norwegian University of Science and Technology, 7491 Trondheim, Norway; Department of Mathematics and Statistics, University of Helsinki, 00014 Helsinki, Finland; Department of Mathematical Sciences and Biocenter Oulu, University of Oulu, 90014 Oulu, Finland

## Abstract

**Key message:**

**A novel reparametrization-based INLA approach as a fast alternative to MCMC for the Bayesian estimation of genetic parameters in multivariate animal model is presented**.

**Abstract:**

Multi-trait genetic parameter estimation is a relevant topic in animal and plant breeding programs because multi-trait analysis can take into account the genetic correlation between different traits and that significantly improves the accuracy of the genetic parameter estimates. Generally, multi-trait analysis is computationally demanding and requires initial estimates of genetic and residual correlations among the traits, while those are difficult to obtain. In this study, we illustrate how to reparametrize covariance matrices of a multivariate animal model/animal models using modified Cholesky decompositions. This reparametrization-based approach is used in the Integrated Nested Laplace Approximation (INLA) methodology to estimate genetic parameters of multivariate animal model. Immediate benefits are: (1) to avoid difficulties of finding good starting values for analysis which can be a problem, for example in Restricted Maximum Likelihood (REML); (2) Bayesian estimation of (co)variance components using INLA is faster to execute than using Markov Chain Monte Carlo (MCMC) especially when realized relationship matrices are dense. The slight drawback is that priors for covariance matrices are assigned for elements of the Cholesky factor but not directly to the covariance matrix elements as in MCMC. Additionally, we illustrate the concordance of the INLA results with the traditional methods like MCMC and REML approaches. We also present results obtained from simulated data sets with replicates and field data in rice.

## Introduction

Estimation of variance components and associated breeding values is an important topic in classic (e.g., Piepho et al. 2008; Oakey et al. 2006; Bauer et al. 2006) and in Bayesian (e.g., Wang et al. 1993; Blasco 2001; Sorensen and Gianola 2002; Mathew et al. 2012) single-trait mixed model context. Similarly, multi-trait models have been proposed in both settings (e.g., Bauer and Léon 2008; Thompson and Meyer 1986; Korsgaard et al. 2003; Van Tassell and Van Vleck 1996; Hadfield 2010). Multi-trait analyses can take into account the correlation structure among all traits and that increases the accuracy of evaluation. However, this gain in accuracy is dependent on the absolute difference between the genetic and residual correlation between the traits (Mrode and Thompson 2005). This evaluation accuracy will increase as the differences between these correlations become high (Schaeffer 1984; Thompson and Meyer 1986). Persson and Andersson (2004) compared single-trait and multi-trait analyses of breeding values and they showed that multi-trait predictors resulted in a lower average bias than the single-trait analysis. Estimation of genetic and residual covariance matrices are the main challenging problem in multi-trait analysis in mixed model framework. However, in Bayesian analysis of multi-trait animal models, inverse-Wishart distribution is the common choice as the prior distribution for those unknown covariance matrices. The use of inverse-Wishart prior distribution for covariance matrix guarantees that the resulting covariance matrix will be positive definite (that is, invertible). However, the use of inverse-Wishart prior distribution is quite restrictive, because then one gives same degrees of freedom for all components in the covariance matrix (Barnard et al. 2000). Moreover, it is often difficult to suggest prior distributions that can be used for common situations. Matrix decomposition approach presented in this paper assigns independent priors for elements in the Cholesky factor.

Markov Chain Monte Carlo (MCMC) methods are a popular choice for Bayesian inference of animal models (Sorensen and Gianola 2002). Often, inference using MCMC methods is challenging for a non-specialist. Although there are various packages available for Bayesian inference which are based on MCMC methods (e.g., MCMCglmm, Hadfield 2010; BUGS, Lunn et al. 2000; Stan, Stan Development Team 2014), most of these packages are not easy to use and computationally expensive. Among these packages, MCMCglmm seems to be easy to implement and computationally inexpensive. As an alternative to MCMC methods one can use the recently implemented non-sampling-based Bayesian inference method, Integrated Nested Laplace Approximation (INLA, Rue et al. 2009). INLA methodology is comparatively easy to implement, but less flexible than MCMC methods (Holand et al. 2013).

Canonical transformation is a common matrix decomposition technique in multi-trait animal models to simultaneously diagonalize the genetic covariance matrix and make residual covariance matrix to identity matrix (see e.g., Ducrocq and Chapuis 1997). After transformation, best linear unbiased prediction (BLUP) values can be calculated independently for each trait using univariate animal model and then back transformed to obtain benefits of multi-trait analysis. However, common requirement in canonical transformation is that covariance matrices need to be known before the transformation. Therefore, it cannot be applied for variance component estimation—with unknown genetic and residual covariance matrices. Here, as an improvement, we introduce another kind of decomposition approach, where elements of the transformation matrix are estimated simultaneously together with the other mixed model parameters allowing us to apply this transformation also for the case of variance component estimation. This kind of modified Cholesky decomposition approach is required to perform multi-trait analysis in INLA (see Bøhn 2014). The closely related decomposition approach has been presented in Pourahmadi (1999, 2000, 2011) and Gao et al. (2015). Also our approach is somewhat related to factor analytic (FA) models (e.g. Meyer 2009; Cullis et al. 2014) and which was first introduced in a breeding context by Piepho (1997, 1998).

In this paper, we illustrate this approach to estimate genetic and residual covariance matrices with INLA and compare the obtained estimates with those from REML (Patterson and Thompson 1971) and MCMC approaches using simulated and real data sets. With the recent development of new low-cost high-throughput DNA sequencing technologies, it is now possible to obtain thousands of single nucleotide polymorphism (SNP) markers covering the whole genome, at the same time, in many animal and plant breeding programs often the detailed pedigree information is available. So we present results obtained using the marker data (real dataset) along with estimates obtained using pedigree information (simulated data) in this study. We also outline a more simple approach to simulate correlated traits based on the additive relationship matrix.

## Models and methods

### Model

We consider the multi-trait mixed model by Henderson and Quaas (1976). Let vector ${\mathbf {y}}_{1}$ represent the $n_{1}$ observations for trait 1, ${\mathbf {y}}_{2}$ represent the $n_{2}$ observations for trait 2 and ${{\mathbf {y}}_{n}}$ represent the $n_{n}$ observations for trait *n*. Then the multi-trait mixed linear model for *n* traits can be written as:

1 $$\begin{aligned} {\mathbf {y}}_i={{\mathbf {X}}_{i}}{\varvec{\beta }}_{i}+{\mathbf {Z}}_i{\mathbf {u}}_i+{{\varvec{\epsilon }}_{i}}, \quad i=1,2\ldots ,n \end{aligned}$$

${\varvec{\beta }}_{{i}}$ is a vector of fixed effects associated with trait $i$, ${{\mathbf {u}}_{i}}$ is a vector of random additive genetic effects associated with trait $i$, ${{\varvec{\epsilon }}_{i}}$ is a vector of error terms, which are independently normally distributed with mean zero and variance $\sigma ^2_{e}$. Moreover, ${{\mathbf {X}}_{i}}$ and ${{\mathbf {Z}}_{i}}$ are known incidence matrices for the fixed effects and the random effects for the trait $i$, respectively. Then, the multi-trait mixed model for $n$ traits can be represented as follows:

2 $$\begin{aligned} \begin{bmatrix} {\mathbf {y}}_{1} \\ {\mathbf {y}}_{2} \\ {\mathbf {.}}\\ {\mathbf {.}}\\ {\mathbf {y}_{n}}\\ \end{bmatrix} = \begin{bmatrix} {\mathbf {X}}_{1}&{\mathbf {0}}&{\mathbf {.}}&.&{\mathbf {0}}\\ {\mathbf {0}}&{\mathbf {X}}_{2}&.&.&{\mathbf {0}} \\ .&.&.&.&.\\ .&.&.&.&.\\ {\mathbf {0}}&{\mathbf {0}}&.&.&{{\mathbf {X}}_{n}}\\ \end{bmatrix} \begin{bmatrix} {\varvec{\beta }}_{1}\\ {\varvec{\beta }}_{2} \\ .\\ .\\ {\varvec{\beta }}_{n}\\ \end{bmatrix}+ \begin{bmatrix} {\mathbf {Z}}_{1}&{\mathbf {0}}&.&.&{\mathbf {0}}\\ {\mathbf {0}}&{\mathbf {Z}}_{2}&.&.&{\mathbf {0}} \\ .&.&.&.&.\\ .&.&.&.&.\\ {\mathbf {0}}&{\mathbf {0}}&.&.&{\mathbf {Z}_{n}}\\ \end{bmatrix} \begin{bmatrix} {\mathbf {u}}_{1}\\ {\mathbf {u}}_{2} \\ .\\ .\\ {\mathbf {u}_{n}} \\ \end{bmatrix} + \begin{bmatrix} {\varvec{\epsilon }}_{1}\\ {\varvec{\epsilon }}_{2} \\ .\\ .\\ {\varvec{\epsilon} _{n}} \\ \end{bmatrix} \end{aligned}$$

Let $${\varvec{\beta }}=[{\varvec{\beta }}^{\prime }_{1},{\varvec{\beta }}^{\prime }_{2}\ldots {\varvec{\beta }}^{\prime }_{n}]^{\prime},$$$$\mathbf {u=[u^{\prime}}_{1},\mathbf {u^{\prime}}_{2}\ldots \mathbf {u^{\prime}}_{n}]^{\prime},$$$$\varvec{\epsilon =[\epsilon ^{\prime }}_{1},\varvec{\epsilon ^{\prime }}_{2}\ldots \varvec{\epsilon ^{\prime }}_{n}]^{\prime }$$ and $$\mathbf {y}$$ contains traits $$\mathbf {y}_{1}\mathbf {\ldots }$$$$\mathbf {y}_{n}$$. In our study we considered three correlated traits so $$i = 1, 2, 3$$. Then mixed model equation (MME) for the model (1) is:

3 $$\begin{aligned} \begin{bmatrix} {\mathbf {X^{\prime }R}}^{-1}{\mathbf {X}}&{\mathbf {X^{\prime }R}}^{-1}{\mathbf {Z}} \\ {\mathbf {Z^{\prime }R}}^{-1}{\mathbf {X}}&{\mathbf {Z^{\prime }R}}^{-1}{\mathbf {Z}}+{\mathbf {G}}^{-1} \\ \end{bmatrix} \begin{bmatrix} {\varvec{\beta }}\\ {\mathbf {u}} \end{bmatrix}= \begin{bmatrix} {\mathbf {X^{\prime }R}}^{-1}{\mathbf {y}} \\ {\mathbf {Z^{\prime }R}}^{-1}{\mathbf {y}} \end{bmatrix}. \end{aligned}$$

Here, ${\mathbf {R}}$ and ${\mathbf {G}}$ are covariance matrices associated with the vector ${\varvec{\epsilon }}$ of residuals and vector $\mathbf {u}$ of random effects. If $\mathbf {R}_{0}$ (of order $3 \times 3$) is the residual covariance for the three traits then $\mathbf {R}$ can be calculated as ${\mathbf {R}}={\mathbf {R}}_{0}\otimes {\mathbf {I}}$ (here ‘$\otimes$’ is the Kronecker product of two matrices and $\mathbf {I}$ is the identity matrix). Similarly, the genetic covariance matrix $\mathbf {G}$ can be calculated as ${\mathbf {G}}={\mathbf {G}}_{0}\otimes {\mathbf {A}}$. Here, $\mathbf {A}$ is the additive genetic relationship matrix (p. 763 in Lynch and Walsh 1998) and $\mathbf {G}_{0}$ is a $3 \times 3$ additive genetic (co)variance matrix. For the Bayesian inference with MCMCglmm package using model (1) one need to specify the conditional distribution for the data ($\mathbf {y}$) and prior distribution for the unknown parameters. So the conditional distribution of data $\mathbf {y}$, given the parameters assumed to follow a multivariate normal distribution:

4 $$\begin{aligned} {\mathbf {y}}|{\varvec{\beta }},{\mathbf {u}},{\mathbf {R}}_{0}\sim {\mathcal {MVN}} ({\mathbf {X}}{\varvec{\beta }}+{\mathbf {Z}}{\mathbf {u}}, {\mathbf {R}}_{0}\otimes {\mathbf {I}}). \end{aligned}$$

The additive genetic effects (${\mathbf {u}}_{i}`s$) were assigned multivariate normal distributions with a mean vector of zeros, $\mathbf 0$, as:

5 $$\begin{aligned} {\mathbf {u}}|{\mathbf {G}_{0}},{\mathbf {A}}\sim {\mathcal {MVN}} (\mathbf {0},\mathbf {G}_{0}\otimes \mathbf {A}), \end{aligned}$$

and the residuals (${\varvec{\epsilon }}_{i}`s$) were assumed to follow,

6 $$\begin{aligned} {\varvec{\epsilon }}|{\mathbf {R}}_{0}\sim {\mathcal {MVN}}(\mathbf {0},\mathbf {R}_{0}\otimes \mathbf {I}), \end{aligned}$$

where $\mathbf I$ is an identity matrix. In Bayesian analysis fixed effects also have a prior and here ${\varvec{\beta }}$ was assigned a vague, large-variance Gaussian prior distribution.

### Reparametrization of trivariate animal model in INLA

Steinsland and Jensen (2010) showed that animal models are latent Gaussian Markov random field (GMRF) models with a sparse precision matrix (inverse of the additive relationship matrix, $\mathbf {A}^{-1}$), and can be analyzed in INLA framework. Mathew et al. (2015), Larsen et al. (2014) and Holand et al. (2013) used INLA for Bayesian inference of univariate animal models, while in a recent study, Bøhn (2014) showed how to analyze a bivariate animal model using INLA. Unlike MCMCglmm and ASReml-R (Butler et al. 2007), analysis of multivariate animal model is not straightforward in R-INLA. For multivariate inference in INLA, we first assumed a trivariate distribution as a set of univariate Gaussian distributions, then we used the multiple likelihood feature in INLA and the recently implemented ‘copy’ feature (Martins et al. 2013) to fit our trivariate animal model with separate likelihoods (but which share few common parameters). The ‘copy’ feature in INLA allows us to estimate dependency parameters between traits. For the INLA analysis of the trivariate animal model we can reparametrize our model for the observation vector $\mathbf {y}$ as follows:

7 $$\begin{aligned} {\mathbf {y}}_{1}&={\mathbf {a}}_{1} + {\mathbf {e}}_{1} \nonumber \\ {\mathbf {y}}_{2}&=\kappa _{1,2}{\mathbf {a}}_{1} +{\mathbf {a}}_{2} +\alpha _{1,2}{\mathbf {e}}_{1}+{\mathbf {e}}_{2}\nonumber \\ {\mathbf {y}}_{3}&=\kappa _{1,3}{\mathbf {a}}_{1} +\kappa _{2,3}{\mathbf {a}}_{2}+ {\mathbf {a}}_{3} +\alpha _{1,3}{\mathbf {e}}_{1}+\alpha _{2,3}{\mathbf {e}}_{2}+{\mathbf {e}}_{3} \end{aligned}$$

Here, $\mathbf {y}_{1}, \mathbf {y}_{2}, \mathbf {y}_{3}$ are the traits and ${\mathbf {\kappa }}_{i,j}$ defines the dependency between additive effects (${\mathbf {a}}_{i}`s$), moreover, ${\mathbf {\alpha }}_{i,j}$ defines the dependency between the error terms (${\mathbf {e}}_{i}`s$). For the Bayesian inference one needs to assign prior distribution for the unknown parameters. The additive genetic effects (${\mathbf {a}}_{i}`s$) for each trait were assigned multivariate normal distributions with a mean vector of zeros, $\mathbf {0}$, as:

$$\begin{aligned} {\mathbf {a}}_{i}|{\mathbf {\sigma }}^2_{a_{i}}\sim {\mathcal {MVN}}({\mathbf {0}},{\mathbf {A}}{\mathbf {\sigma }}^2_{a_{i}}), \end{aligned}$$

whereas the residuals (${\mathbf {e}}_{i}`s$) were assumed to follow a multivariate normal distribution as follows:

$$\begin{aligned} {\mathbf {e}}_{i}|{\mathbf {\sigma }}^2_{{e}_{i}}\sim {\mathcal {MVN}}(\mathbf {0},{\mathbf {I}}{\mathbf {\sigma }}^2_{e_{i}}), \end{aligned}$$

where $\mathbf I$ is an identity matrix. The hyperparameters (${\mathbf {\sigma }}^2_{a_{i}},{\mathbf {\sigma }}^2_{e_{i}}$) were assigned inverse-Gamma prior (0.5, 0.5) distributions and the dependency parameters ($\kappa _{i,j},\alpha _{i,j}$) were assumed to follow Gaussian distributions with mean 0 and variance 10. Thus we define the observation vector $\mathbf {y}$ for the trivariate animal model as:

8 $$\begin{aligned} {\mathbf {y}}={\mathbf {u}}+{\varvec{\epsilon }}. \end{aligned}$$

Here ${\mathbf {u}}={{\mathbf {W}}_{a}{\mathbf{a}}}$, is the additive genetic term and ${\varvec{\epsilon }}={{\mathbf {W}}_{e}{\mathbf{e}}}$, is the residual term. Moreover, Cholesky factor

$$\begin{aligned} {\mathbf {W}_{a}} =\begin{bmatrix} 1&0&0 \\ \kappa _{1,2}&1&0 \\ \kappa _{1,3}&\kappa _{2,3}&1 \end{bmatrix}\end{aligned}\otimes \mathbf{I} =\begin{aligned}\begin{bmatrix} \mathbf {I}&\mathbf {0}&\mathbf {0} \\ \kappa _{1,2}\mathbf{I}&\mathbf {I}&\mathbf {0} \\ \kappa _{1,3}\mathbf{I}&\kappa _{2,3}\mathbf{I}&\mathbf {I} \end{bmatrix} \end{aligned}$$

and

$$\begin{aligned} {\mathbf {a}}= \begin{bmatrix} {\mathbf {a}}_1 \\ {\mathbf {a}}_2\\ {\mathbf {a}}_3 \end{bmatrix}\sim {\mathcal {MVN}}\left (\begin{bmatrix} {\mathbf {0}} \\ {\mathbf {0}} \\ {\mathbf {0}} \end{bmatrix}, \begin{bmatrix} {\mathbf {\sigma }^2_{{a}_{1}}{\mathbf {A}}}&\mathbf {0}&\mathbf {0} \\ \mathbf {0}&{\mathbf {\sigma }^2_{{a}_{2}}{\mathbf{A}}}&\mathbf {0} \\ \mathbf {0}&\mathbf {0}&{\mathbf {\sigma }^2_{{a}_{3}}{\mathbf {A}}} \end{bmatrix}\right). \end{aligned}$$

Here, $\mathbf {a}_1,\mathbf {a}_2$ and $\mathbf {a}_3$ are the additive effects for each traits in the reparametrized scale. Moreover, $\mathbf {A}$ is additive relationship matrix calculated from the pedigree information and $\mathbf {\sigma }^2_{{a}_{i}}$, $i = 1, 2, 3$ are the additive genetic variances for each trait. Hence,

$$\begin{aligned} {\mathbf {u}} \sim {\mathcal {MVN}}\left (\begin{bmatrix} \mathbf {0} \\ \mathbf {0} \\ \mathbf {0} \end{bmatrix},{{\mathbf {W}}_a\Sigma _{X_a}{\mathbf {W}}^{T}_{a}}\right ). \end{aligned}$$

Here,

$$\begin{aligned} {\mathbf {W}_a\Sigma _{X_a}{\mathbf{W}}^{T}_{a}}=\begin{bmatrix} \mathbf {I}&\mathbf {0}&\mathbf {0} \\ \kappa _{1,2}\mathbf{I}&\mathbf {I}&\mathbf {0} \\ \kappa _{1,3}\mathbf{I}&\kappa _{2,3}\mathbf{I}&\mathbf {I} \end{bmatrix} \begin{bmatrix} {\mathbf {\sigma }}^2_{{a}_{1}}{\mathbf {A}}&\mathbf {0}&\mathbf {0} \\ \mathbf {0}&{\mathbf {\sigma }}^2_{{a}_{2}}{\mathbf {A}}&\mathbf {0} \\ \mathbf {0}&\mathbf {0}&{\mathbf {\sigma }}^2_{{a}_{3}}{\mathbf {A}} \end{bmatrix} \begin{bmatrix} \mathbf {I}&\kappa _{1,2}\mathbf {I}&\kappa _{1,3}\mathbf {I} \\ \mathbf {0}&\mathbf {I}&\kappa _{2,3}\mathbf {I} \\ \mathbf {0}&\mathbf {0}&\mathbf {I} \end{bmatrix} \end{aligned}$$

9 $$\begin{aligned} = \begin{bmatrix} {\mathbf {\sigma }}^2_{{a}_{1}}{\mathbf {A}}&\kappa _{1,2}{\mathbf {\sigma }}^2_{{a}_{1}}{\mathbf {A}}&\kappa _{1,3}{\mathbf {\sigma }}^2_{{a}_{1}}{\mathbf {A}}\\ \kappa _{1,2}{\mathbf {\sigma }}^2_{{a}_{1}}{\mathbf {A}}&\kappa ^2_{1,2}{\mathbf {\sigma }}^2_{{a}_{1}}{\mathbf {A}} +{\mathbf {\sigma }}^2_{{a}_{2}}{\mathbf {A}}&\kappa _{1,3}\kappa _{1,2}{\mathbf {\sigma }}^2_{{a}_{1}}{\mathbf {A}}+\kappa _{2,3}{\mathbf {\sigma }}^2_{{a}_{2}}{\mathbf {A}} \\ \kappa _{1,3}{\mathbf {\sigma }}^2_{{a}_{1}}{\mathbf {A}}&\kappa _{1,2}\kappa _{1,3}{\mathbf {\sigma }}^2_{{a}_{1}}{\mathbf {A}}+\kappa _{2,3}{\mathbf {\sigma }}^2_{{a}_{2}}{\mathbf {A}}&\kappa ^2_{1,3}{\mathbf {\sigma }}^2_{{a}_{1}}{\mathbf {A}}+\kappa ^2_{2,3}{\mathbf {\sigma }}^2_{{a}_{2}}{\mathbf {A}}+{\mathbf {\sigma }}^2_{{a}_{3}}{\mathbf {A}} \end{bmatrix} \end{aligned}$$

and

$$\begin{aligned} \mathbf {\Sigma }_{X_a} = \begin{bmatrix} {\mathbf {\sigma }}^2_{{a}_{1}}{\mathbf {A}}&\mathbf {0}&\mathbf {0} \\ \mathbf {0}&{\mathbf {\sigma }}^2_{{a}_{2}}{\mathbf {A}}&\mathbf {0} \\ \mathbf {0}&\mathbf {0}&{\mathbf {\sigma }}^2_{{a}_{3}}{\mathbf {A}} \end{bmatrix} \end{aligned}$$

is the additive genetic covariance matrix for the traits in the transformed scale. Thus, the additive genetic effects (${\mathbf {u=W}_{{a}}{\mathbf{a}}}$) follow a multivariate normal distribution (Eq. 5) with a mean vector of zeros, $\mathbf 0$, as:

10 $$\begin{aligned} {\mathbf {u}}|{\mathbf {G}}_{0},{\mathbf {A}}\sim {\mathcal {MVN}} (\mathbf {0},{\mathbf {G}}_{0}\otimes {\mathbf {A}}). \end{aligned}$$

Here, $\mathbf {G}_{0}$ is the additive genetic (co)variance matrix. Similarly

11 $$\begin{aligned} {\varvec{\epsilon }}\sim {\mathcal {MVN}}\left(\begin{bmatrix} \mathbf {0} \\ \mathbf {0} \\ \mathbf {0} \end{bmatrix},\begin{bmatrix} {\mathbf {\sigma }}^2_{{e}_{1}}{\mathbf {I}}&\alpha _{1,2}{\mathbf {\sigma }}^2_{{e}_{1}}{\mathbf {I}}&\alpha _{1,3}{\mathbf {\sigma }}^2_{{e}_{1}}{\mathbf {I}} \\ \alpha _{1,2}{\mathbf {\sigma }}^2_{{e}_{1}}{\mathbf {I}}&\alpha ^2_{1,2}{\mathbf {\sigma }}^2_{{e}_{1}}{\mathbf {I}}+{\mathbf {\sigma }}^2_{{e}_{2}}{\mathbf {I}}&\alpha _{1,2}\alpha _{1,3}{\mathbf {\sigma }}^2_{{e}_{1}}{\mathbf {I}}+\alpha _{2,3}{\varvec{\sigma }}^2_{e_2}{\mathbf {I}}\\ \alpha _{1,3}{\mathbf {\sigma }}^2_{{e}_{1}}{\mathbf {I}}&\alpha _{1,2}\alpha _{1,3}{\mathbf {\sigma }}^2_{{e}_{1}}{\mathbf {I}}+\alpha _{2,3}{\mathbf {\sigma }}^2_{{e}_{2}}{\mathbf {I}}&\alpha ^2_{1,3}{\mathbf {\sigma }}^2_{{e}_{1}}{\mathbf {I}}+\alpha ^2_{2,3}{\mathbf {\sigma }}^2_{{e}_{2}}{\mathbf {I}}+{\mathbf {\sigma }}^2_{{e}_{3}}{\mathbf {I}} \end{bmatrix}\right). \end{aligned}$$

Here we have independent error terms (${\mathbf {e}}_i` s$) for each trait, so the covariance matrix $\mathbf {I}$ is an identity matrix. Hence, the residuals (${\varvec{\epsilon }}$) follow a multivariate normal distribution (Eq. 6) with a mean vector of zeros, $\mathbf {0}$, as follows:

12 $$\begin{aligned} {\varvec{\epsilon }}|{\mathbf {R}}_{0}\sim {\mathcal {MVN}}(\mathbf {0},{\mathbf {R}}_{0}\otimes {\mathbf {I}}). \end{aligned}$$

Here, $\mathbf {R}_{0}$ is the residual genetic (co)variance matrix.

To extend this method for more than three traits (say, *n* traits) can be done by modifying the terms of Eq. (9), so that the additive genetic Cholesky factor ${\mathbf {W}_{a}}$ is a Kronecker product of $n\times n$ lower triangular matrix with **I** and ${\mathbf {\Sigma }}_{{X}_{a}}$ is the additive genetic block matrix containing *n* blocks. For example, number of dependency parameters required for a $4 \times 4$ Cholesky factor is already $n(n-1)/2=4\times 3/2=6$.

As an additional supplementary material we provide the R scripts we used for the INLA analysis.

### Back transformation in INLA

INLA analysis returns the marginal posterior distributions of the hyperparameters (${\mathbf {\sigma }}^2_{a_{i}`s},{\mathbf {\sigma }}^2_{e_{i}`s}$) and the dependency parameters ($\kappa_{i,j}`s,\alpha _{i,j}`s$) for the reparametrized model (Eq. 7). So one need to perform the back transformation after the INLA analysis in order to obtain (co)variance components in the original scale. Let ${\mathbf {\sigma }}^2_{u_{i}}$, ${\mathbf {\sigma }}^2_{\epsilon _{i}}$, where $i = 1, 2, 3$ and ${\mathbf {\sigma }}_{u_{ij}}$, ${\mathbf {\sigma }}_{\epsilon _{ij}}$, where $i,j = 1, 2, 3$ be the (genetic and residual) variance and (genetic and residual) covariance components, respectively, in the original scale. First, calculate the approximated posterior marginal distribution for the hyperparameters (${\mathbf {\sigma }}^2_{a_{i}`s},{\mathbf {\sigma }}^2_{e_{i}`s}$) and the dependency parameters ($\kappa_{i,j}`s,\alpha _{i,j}`s$) by sampling from their joint distribution using the ‘inla.hyperpar.sample’ (Martins et al. 2013) function. Then, following Eq. (9) the genetic variance components can be calculated using the posterior distributions as, ${\mathbf {\sigma }}^2_{{u}_{1}}={\mathbf {\sigma }}^2_{{a}_{1}}$, ${\mathbf {\sigma }}^2_{{u}_{2}}=\kappa ^2_{1,2}\sigma ^2_{{a}_1} +{\mathbf {\sigma }}^2_{{a}_2}$ and ${\mathbf {\sigma }}^2_{{u}_{3}}={\mathbf {\kappa }}^2_{1,3}\sigma ^2_{{a}_1}+{\mathbf {\kappa }}^2_{2,3}\sigma ^2_{{a}_2}+{\mathbf {\sigma }}^2_{{a}_3}$. Similarly the genetic covariance components can be obtained as, ${\mathbf {\sigma }}_{{u}_{12}}={\mathbf {\kappa }}_{1,2}\sigma ^2_{{a}_1}$, ${\mathbf {\sigma }}_{{u}_{13}}={\mathbf {\kappa }}_{1,3}\sigma ^2_{{a}_1}$, and ${\mathbf {\sigma }}_{u_{23}}={\mathbf {\kappa }}_{1,2}\kappa _{1,3}\sigma ^2_{{a}_1}+{\mathbf {\kappa }}_{2,3}\sigma ^2_{{a}_2}$. The same procedure can be used to calculate the residual (co)variance components using Eq. (11). R scripts for the back transformation can be found in the supplementary material.

## Example analyses

### Simulated dataset with high heritability

To validate our new algorithm we developed a simulated pedigree data. In this, we considered a base population of 50 unrelated lines, wherein each of the 25 seed parents were mated with 25 pollen donors resulting in total 625 individuals (in total 675 individuals, including the base population). Additive genetic relationship matrix ($\mathbf {A}$) was calculated from the pedigree information. In our current study, we simulated three quantitative traits by summing up the additive genetic effects $\mathbf {a}$ and the noise $\mathbf {e}$. Thus, the vector of phenotypic observations (three traits) was calculated as:

$$\begin{aligned} {\mathbf {y}}={\mathbf {a}}+{\mathbf {e}}. \end{aligned}$$

Here the vectors $\mathbf {a}$, $\mathbf {e}$ were drawn from ${\mathcal {MVN}}({\mathbf {0}},{\mathbf {G}}_{0}\otimes {\mathbf {A}})$ and ${\mathcal {MVN}}({\mathbf {0}},{\mathbf {R}}_{0}\otimes {\mathbf {I}})$, respectively. In order to simulate correlated traits with relatively high heritability ($h^2 \geqslant 0.5$), we used

13 $$\begin{aligned} {\mathbf {G}}_{0} = \begin{bmatrix} 1.0&0.4&0.6 \\ 0.4&1.5&0.9 \\ 0.6&0.9&2.5 \end{bmatrix},\quad {\mathbf {R}}_{0} = \begin{bmatrix} 1.0&0.5&0.5 \\ 0.5&1.0&0.5 \\ 0.5&0.5&1.0 \end{bmatrix} \end{aligned}$$

as the genetic covariance matrix and the residual covariance matrix between the three traits. The three simulated traits had heritabilities $\approx 0.50, 0.60$ and 0.70, respectively. Let ${\mathbf {G}}={\mathbf {G}}_{0}\otimes {\mathbf {A}}$ and ${\mathbf {R}}={\mathbf {R}}_{0}\otimes {\mathbf {I}}$, then we used the Cholesky decomposition of the covariance matrices $\mathbf {G}$ and $\mathbf {R}$ to draw samples from the multivariate normal distribution. Hence, the random additive effect $\mathbf {a}$ was calculated as ${\mathbf {a}}={\mathbf {Pz}_{{a}}}$, where ${\mathbf {z}_{{a}}\sim {\mathcal {MVN}}(\mathbf {0},\mathbf {I})}$ and $\mathbf {P}$ is the Cholesky factor $\mathbf {PP^{\prime }=G}$; whereas, the residuals $\mathbf {e}$ was calculated as ${\mathbf {e}}={\mathbf {Tz}_{{e}}}$, where ${\mathbf {z}_{{e}}\sim {\mathcal {MVN}}(\mathbf {0},\mathbf {I})}$ and $\mathbf {T}$ is the Cholesky factor $\mathbf {TT^{\prime }=R}$.

### Simulated dataset with low heritability

We also analyzed another simulated correlated dataset with low heritability ($h^2\approx 0.2$) and negative covariances between the traits, in order to show how these methods perform when the heritability is relatively low. To simulate the dataset we considered the same pedigree information from the high heritability dataset but, with different covariance matrices. For the simulation we considered

$$\begin{aligned} {\mathbf {G}}_{0} = \begin{bmatrix} 5&-2&3 \\ -2&7&4\\ 3&4&10\end{bmatrix},\quad {\mathbf {R}}_{0} = \begin{bmatrix} 20&-5&1 \\ -5&28&3 \\ 1&3&35 \end{bmatrix} \end{aligned}$$

as the genetic and residual covariance matrices, respectively. The correlated phenotypes were simulated as explained before and the three traits had heritabilities $\approx$0.20, 0.20 and 0.22, respectively.

### Field data

In our study we analyzed the recently published rice (*Oryza sativa*) dataset (Spindel et al. 2015) and we selected three traits, grain yield (YLD), flowering time (FL) and plant height (PH) from 2012 dry season for the analysis. The population was genotyped with 73,147 markers using genotyping-by-sequencing method and we selected 323 lines where both the phenotypic and genotypic informations were available (see  Spindel et al. 2015 for more details). So we used the available marker information for the estimation of genetic (co)variance components and the realized genomic relationship matrix ($\mathbf {M}$) was obtained from the marker information using R-package ‘rrBLUP’ (Endelman 2011). For the real data analysis, we considered the marker data instead of the pedigree information, so in model (1) the vector of random effects ($\mathbf {u}$) were assumed to follow a normal distribution according to Eq. (5) as

14 $$\begin{aligned} {\mathbf {u}}|{\mathbf {G}}_{0},{\mathbf {M}}\sim {\mathcal {MVN}} ({\mathbf {0}},{\mathbf {G}}_{0}\otimes {\mathbf {M}}). \end{aligned}$$

Here, $\mathbf {M}$ is realized genomic relationship matrix calculated from the marker information and $\mathbf {G}_{0}$ is the genetic (co)variance matrix.

## Analyses and results

### Simulated data with replicates

In multi-trait analysis using iterative algorithms, it is often difficult to find suitable starting values for the parameters of interest. However, by performing test-runs using single-trait data one could find suitable starting values for the variance components. The (co)variance components were estimated using MCMCglmm, R-INLA and ASReml-R packages. For MCMC analysis using MCMCglmm package, we considered a total chain length of 50,000 iterations with a burning period of 10,000 iterations. The MCMCglmm package assign inverse-Wishart prior distribution for the random and residual covariance matrices. In our MCMC analysis, we used identity matrix as the scaling matrix of the prior distribution (ones for the variances and zeros for the covariances) assigned for the genetic covariance matrix (${\mathbf {G}}_{0}$) and for the residual covariance matrix (${\mathbf {R}}_{0}$) between the three traits. Moreover, we specified the degree of belief parameter (d) as 1 for the inverse-Wishart prior distribution. By default MCMCglmm uses the scaling matrix values as the starting values. For the REML analysis we used ones as the variances and zeros as the covariances for the genetic covariance matrix (${\mathbf {G}}_{0}$) as the starting values; whereas, for the residual covariance matrix (${\mathbf {R}}_{0}$) we used half of the phenotypic variance matrix of the data as initial values (ASReml-R default). The total computation time for the simulated dataset using MCMCglmm package was around 10 min and INLA took around 4 min, whereas the time for ASReml-R package was around 1 min. The INLA approach we used in the current study was not able to analyze bigger datasets (around 1000 lines), mainly due to the lack of memory on our system. We used a Linux system with 8GB RAM for our calculations. However, it is possible to analyze such large datasets using computers with more memory size or arguably one can use the option ‘inla.remote()’ to run R-INLA on a remote server with more memory size.

**Fig. 1:**
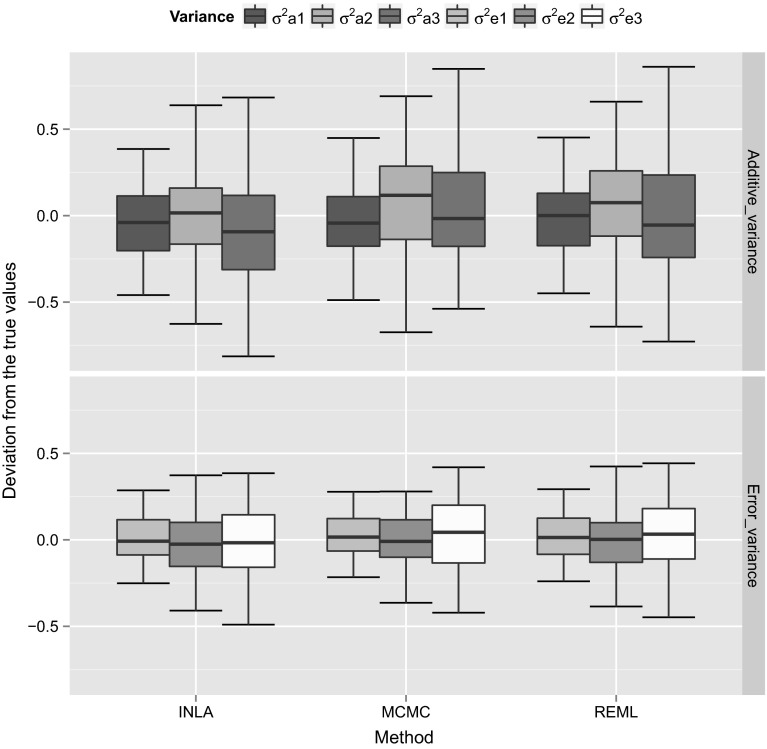
Box plots for the estimation error (difference between the true and estimated values) of the variance components using 50 simulation replicates with the high heritability dataset. Here the *Y*-axis scale corresponds to the difference between the true simulated values and the estimated values, whereas *X*-axis corresponds to different estimation methods

**Fig. 2:**
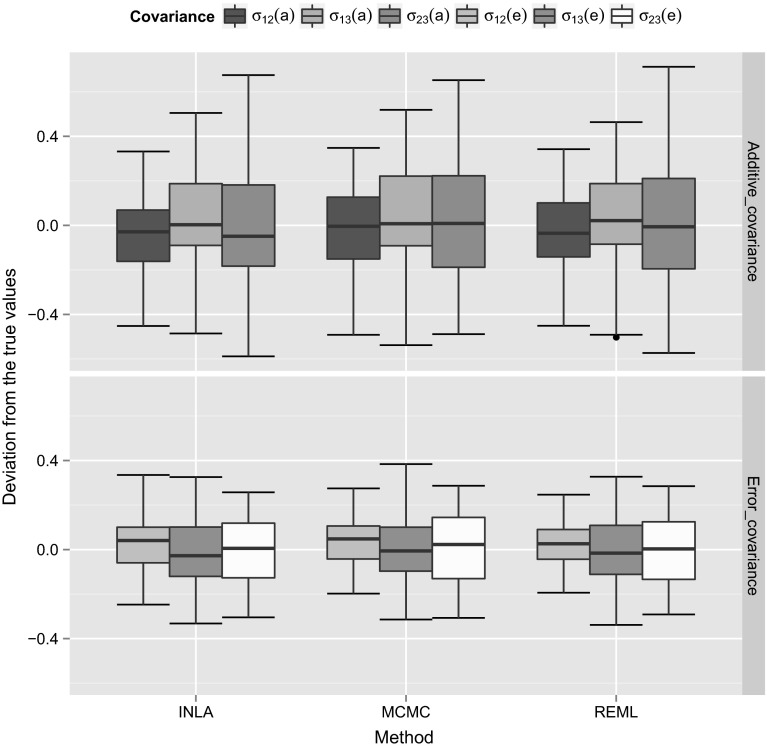
Box plots for the estimation error (difference between the true and estimated values) of the covariance components using 50 simulation replicates with the high heritability dataset. Here the *Y*-axis scale corresponds to the difference between the true simulated values and the estimated values, whereas *X*-axis corresponds to different estimation methods

**Fig. 3:**
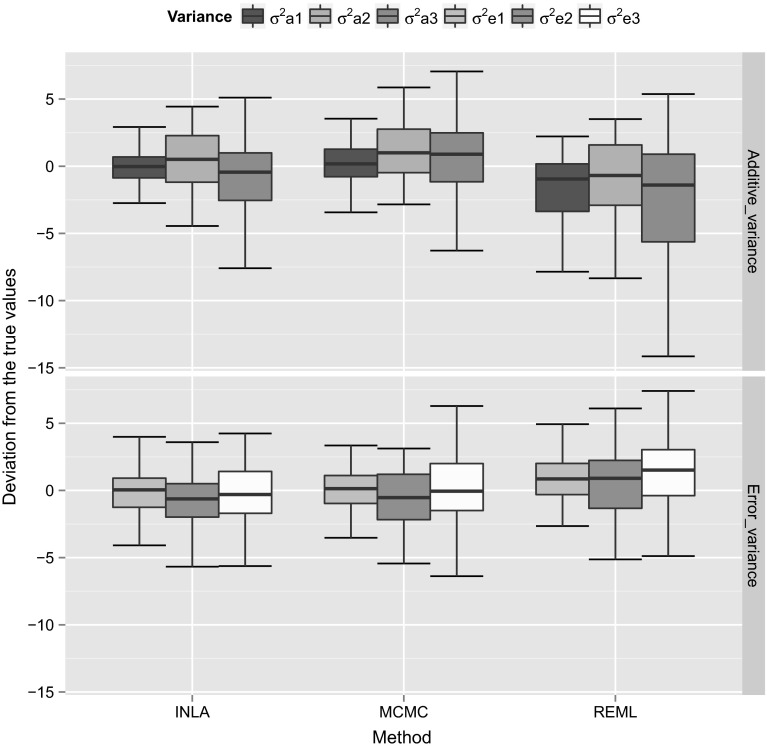
Box plots for the estimation error (difference between the true and estimated values) of the variance components using 50 simulation replicates with the low heritability dataset. Here the *Y*-axis scale corresponds to the difference between the true simulated values and estimated values, whereas *X*-axis corresponds to different estimation methods

**Fig. 4:**
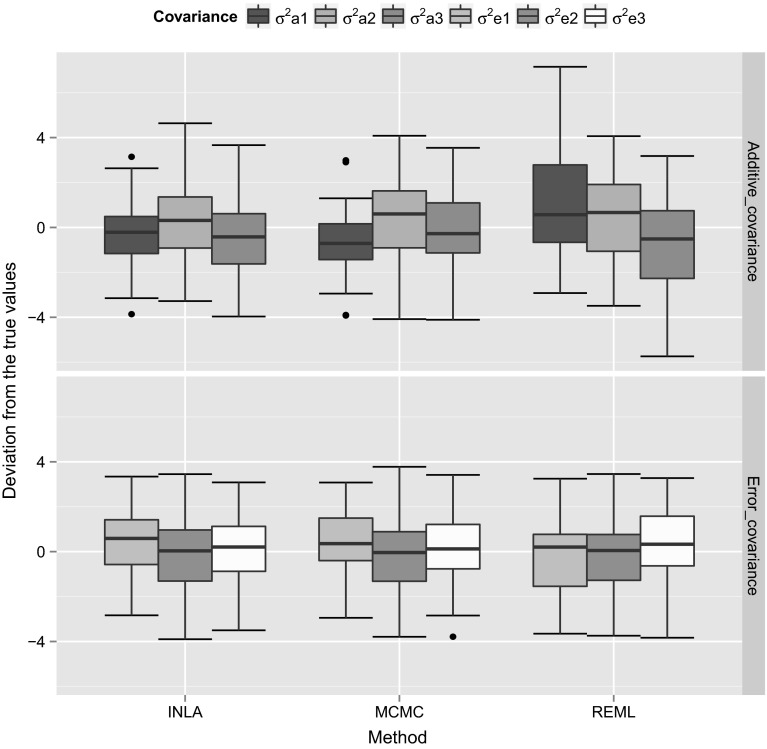
Box plots for the estimation error (difference between the true and estimated values) of the covariance components using 50 simulation replicates with the low heritability dataset. Here the *Y*-axis scale corresponds to the difference between the true simulated values and the estimated values, whereas *X*-axis corresponds to different estimation methods

We used 50 simulation replicates for each simulated dataset to calculate the variance and covariance components using different estimation methods. In order to compare the accuracy of different estimation methods, we calculated the estimation error (difference between the true and estimated values) using 50 simulation replicates for the (co)variance components and then we plotted the box plots for the estimation errors to visualize the estimation accuracy of different methods. We show those box plots for the estimation errors for the variance (Fig. 1) and covariance (Fig. 2) components for the simulated dataset with high heritability. The *Y*-axis scale in those plots corresponds to the differences between the true simulated values and the estimated values, whereas the *X*-axis corresponds to different estimation methods. In order to calculate the estimation errors, for MCMC we used posterior mode, whereas for INLA we used the posterior mean estimates. From Figs. 1 and 2, it can be concluded that, different methods were able to provide similar estimates. We also plotted the box plots for the estimation errors for the variance (Fig. 3) and covariance (Fig. 4) for the dataset with low heritability. However, for the low heritability dataset the MCMC and INLA approaches provided variance estimates closer to true values than the REML method. The narrow-sense heritability estimates for the simulated datasets using 50 simulation replicates are shown in Table 4. Here we did not account the covariances between the traits in order to calculate the heritability. The narrow-sense heritability ($h^2$) was calculated for each trait separately as ${h}^2 = V_a/(V_a+V_e)$, where $V_a$ and $V_e$ are the additive genetic variance and error variance of the particular trait, respectively.

Additionally, instead of covariances we report the estimated genetic and residual correlation coefficients as well as the 95 % empirical confidence intervals (in brackets) for each trait in Table 1. From Table 1 it is clear that the Bayesian methods were able to provide better estimates (closer to the true simulated values) for the additive genetic correlation coefficients than the REML approach with the low heritability dataset. One probable reason could be that the prior influence is higher with the low heritability dataset. We also performed univariate analyses using INLA with the simulated low heritability dataset (see Table 2). Both univariate and multivariate analyses gave very similar results, however in multivariate analysis one can account for and estimate the covariances between the traits.

**Table 1:** Estimated genetic and residual correlation coefficients ($\rho$), between each traits ($T_1$ to $T_3$) for both simulated dataset using REML, INLA and MCMC estimates

Correlation	REML	MCMC	INLA	True
High heritability dataset				
$\rho _{T_{1},T_{2}(a)}$	0.31(0.28, 0.32)	0.32(0.28, 0.33)	0.30(0.27, 0.32)	0.33
$\rho _{T_{1},T_{3}(a)}$	0.41(0.37, 0.43)	0.40(0.37, 0.43)	0.40(0.38, 0.43)	0.38
$\rho _{T_{2},T_{3}(a)}$	0.45(0.44, 0.48)	0.45(0.44, 0.47)	0.47(0.44, 0.48)	0.46
$\rho _{T_{1},T_{2}(e)}$	0.51(0.50, 0.53)	0.52(0.51, 0.53)	0.52(0.51, 0.53)	0.50
$\rho _{T_{1},T_{3}(e)}$	0.48(0.46, 0.50)	0.49(0.46, 0.51)	0.49(0.47, 0.50)	0.50
$\rho _{T_{2},T_{3}(e)}$	0.50(0.48, 0.51)	0.51(0.48, 0.52)	0.50(0.48, 0.52)	0.50
Low heritability dataset				
$\rho _{T_{1},T_{2}(a)}$	−0.19(−0.41, −0.03)	−0.40(−0.50, −0.31)	−0.38(−0.49, −0.29)	−0.34
$\rho _{T_{1},T_{3}(a)}$	0.65(0.64, 0.66)	0.44(0.39, 0.47)	0.47(0.43, 0.51)	0.42
$\rho _{T_{2},T_{3}(a)}$	0.49(0.48, 0.50)	0.43(0.39, 0.44)	0.42(0.40, 0.45)	0.45
$\rho _{T_{1},T_{2}(e)}$	−0.22(−0.24, −0.19)	−0.19(−0.21, −0.17)	−0.20(−0.21, −0.17)	−0.21
$\rho _{T_{1},T_{3}(e)}$	0.02(0.01, 0.04)	0.03(0.01, 0.04)	0.03(0.02, 0.04)	0.04
$\rho _{T_{2},T_{3}(e)}$	0.11(0.09, 0.12)	0.10(0.08, 0.11)	0.10(0.08, 0.11)	0.10

In order to calculate genetic (*a*) and residual (*e*) correlation coefficients, 50 simulation replicates were used and the true simulated values are also given. Additionally, the empirical 95 % confidence intervals for estimates are given in brackets

**Table 2:** The additive genetic variance ($\sigma ^2_{a})$ and the error variance ($\sigma ^2_{e}$) components obtained using a univariate INLA analysis (INLA-U) using the simulated dataset with negative covariance

Variance parameter	INLA-U	INLA-M	True
$\sigma ^2_{a1}$	5.03	4.92	5.00
$\sigma ^2_{a2}$	5.99	6.45	7.00
$\sigma ^2_{a3}$	10.15	10.34	10.00
$\sigma ^2_{e1}$	20.03	20.16	20.00
$\sigma ^2_{e2}$	28.91	28.93	28.00
$\sigma ^2_{e3}$	35.08	35.30	35.00

In order to calculate the INLA estimates, mean of 50 simulation replicates were used. True simulated values and the estimates from the multivariate INLA (INLA-M) analysis are also given

Heritability and breeding values are of great interest to breeders in order to plan an efficient breeding program. In our study, we also calculated the correlation coefficients between the estimated and true breeding values using different estimation methods. We used average over 50 simulation replicates for both datasets to calculate the correlation coefficients. For the high heritability dataset, the correlation coefficients were 0.85, 0.84 and 0.83 for REML, MCMC and INLA methods, respectively. However, for the low heritability dataset the correlation coefficients were relatively low being 0.64, 0.65 and 0.58 for REML, MCMC and INLA, respectively.

### Field data

We chose the same starting values for the simulated data and real data in our REML analysis. For MCMC analysis we used empirical phenotypic variance of each trait as the variances and zeros as the covariance as the scale matrix of the prior distribution, whereas, starting values for other parameters were set randomly. For the REML analysis we chose the same starting values that we used for the simulated dataset. Both REML and Bayesian methods gave similar results in our analysis using real dataset (Table 3). Due to numerical problems caused by the large differences among the traits’ phenotypic variances, before the INLA analysis we standardized each phenotypic vector to zero mean and unit variance, and after the analysis we rescaled the (co)variance components into the original scale. However, for MCMC and REML analysis we used the original scale. Our results showed that there is a negative genetic covariance between the traits plant height (PH) and yield (YLD). Additionally, as expected, the traits days to flowering (FL) and yield (YLD) showed a negative genetic covariance in our analysis. We also calculated the narrow-sense heritability for both datasets and Table 4 summarizes those results. Our narrow-sense heritability estimates for the real dataset are in concordance with the heritability estimates reported by Spindel et al. (2015) for the univariate animal model using REML. The total computation time for real dataset using INLA was around three minutes, whereas the MCMCglmm took around five hours. The main reasons for the expensive computation time with MCMCglmm are, firstly, the realized genomic relationship matrix were calculated outside the package, whereas, for the pedigree information MCMCglmm has built in functions in order to handle the covariance matrix more efficiently. Secondly, the realized genomic relationship matrix from the marker information is more dense than the pedigree-based additive relationship matrix.

**Table 3:** The additive genetic variance ($\sigma ^2_{a}$) and the error variance ($\sigma ^2_{e}$) for the field data obtained from the REML analysis and the posterior mode estimates obtained from the MCMCglmm package along with R-INLA posterior mean estimates are presented

(Co)variance parameter	REML	MCMC	INLA
$\sigma ^2_{{\text{PH}}_{a}}$	22.92	21.85	24.09
$\sigma ^2_{{\text{FL}}_{a}}$	6.50	6.56	6.58
$\sigma ^2_{{\text{YLD}}_{a}}$	34,285.92	35,890.51	40,218.69
$\sigma ^2_{{\text{PH}}_{e}}$	42.34	40.91	43.68
$\sigma ^2_{{\text{FL}}_{e}}$	8.17	8.23	8.47
$\sigma ^2_{{\text{YLD}}_{e}}$	71,698.64	73,983.14	74,211.42
$\sigma _{{\text{PH}},{\text{FL}}(a)}$	4.57	4.48	4.60
$\sigma _{{\text{PH}},{\text{YLD}}(a)}$	−285.05	−237.21	−263.99
$\sigma _{{\text{FL}},{\text{YLD}}(a)}$	−158.50	−120.01	−161.97
$\sigma _{{\text{PH}},{\text{FL}}(e)}$	2.41	2.78	2.57
$\sigma _{{\text{PH}},{\text{YLD}}(e)}$	106.36	76.83	83.21
$\sigma _{{\text{FL}},{\text{YLD}}(e)}$	−38.75	−23.82	−44.09

The additive genetic covariance ($\sigma _{T_{i},T_{j}(a)}$) and the error covariance ($\sigma _{T_{i},T_{j}(e)}$) between each pair of three quantitative traits (PH, FL, YLD) are also shown

**Table 4:** Narrow-sense heritability estimates ($h^2$) for the simulated datasets (averaged over 50 simulation replicates) and the real dataset with the different estimation methods

	REML	MCMC	INLA	True
High heritability dataset				
Trait1	0.49	0.49	0.49	0.50
Trait2	0.61	0.62	0.61	0.60
Trait3	0.71	0.72	0.71	0.71
Low heritability dataset				
Trait1	0.15	0.21	0.20	0.20
Trait2	0.17	0.23	0.22	0.20
Trait3	0.17	0.23	0.21	0.22
Real data				Spindel et al. (2015)
PH	0.35	0.35	0.35	0.35
FL	0.44	0.44	0.44	0.43
YLD	0.32	0.32	0.35	0.32

Posterior mean estimates of R-INLA and posterior modes from MCMCglmm were used for the calculation. Additionally, the true heritability estimates for the simulated dataset and the heritability estimates reported by Spindel et al. (2015) are also shown

## Discussion

Multi-trait analysis of mixed models tend to be powerful and provide more accurate estimates than the single-trait analysis because the former method can take into account the underlying correlation structure found in a multi-trait dataset. However, Bayesian and non-Bayesian inference of multi-trait mixed model analysis are complex and computationally demanding. In this study, we explained how to do Bayesian inference of a multivariate animal model using recently developed INLA and the counter part MCMC, while comparing the results with the commonly used REML estimates. Our results show that reparametrization-based INLA approach can be used as a fast alternative to MCMC methods for the Bayesian inference of multivariate animal model. The reparametrization approach, that was here applied for INLA analysis, can be used also more generally together with other tools to speed up the multi-trait animal model computations.

Drawback of the reparametrization-based approach is that priors are assigned for elements in the Cholesky factor instead of the original covariance matrix. Thus, here it is not possible to make a direct comparison between the MCMC and INLA results due to the differences in the prior distributions, however, it is possible to compare both approaches if we choose the same prior distributions. For the MCMC analysis we used inverse-Wishart distributions for the covariance matrices; whereas, for INLA we used Gaussian prior distribution for the elements in the Cholesky factor (i.e., dependence parameters) (${\mathbf {\kappa }}_{ij}`s,{\mathbf {\alpha }}_{ij}`s$) and inverse-Gamma distribution for the decomposition variance components (${\mathbf {\sigma }}^2_{a_{i}`s},{\mathbf {\sigma }}^2_{e_{i}`s}$). Our results show that the REML estimates are in concordance with MCMCglmm and INLA. We want to emphasize that in our examples, the analyzed data sets were large and we did not encounter any problems. In general, identifiability is a problem in mixed model analyses with small data (Mathew et al. 2012). However, Bayesian methods are in better positions because they can at least find such problems (that posterior distribution has multiple modes) more easily than REML (which provides a single point-estimate).

Nowadays, molecular markers are widely used in animal and plant breeding programs as a valuable tool for genetic improvement. Therefore, we also showed how to estimate genetic parameters in a multivariate animal model using molecular marker information with the reparametrization-based INLA approach and frequentist framework. Finally, our results imply that the reparametrization-based INLA approach can be used as a fast alternative to MCMC methods in order to estimate genetic parameters with a multivariate animal model using pedigree information as well as with molecular marker information.

### Author contribution statement

BM, AH, PK, JL and MS were involved in the conception and design of the study. BM performed the simulations and preprocessing of the data. BM and AH implemented the method, and performed the statistical analyses. BM drafted the manuscript. BM, AH, JL and MS participated in the interpretation of results. All the authors critically revised the manuscript.
